# Impact of blood sampling time deviations on vancomycin therapeutic drug monitoring and clinical decision-making

**DOI:** 10.3389/fmed.2026.1862353

**Published:** 2026-07-01

**Authors:** Mingxin Guo, Han Wang, Sang Xu, Zhiqiang Hu, Yichun Xu

**Affiliations:** 1 Department of Pharmacy, The Affiliated Yixing Hospital of Jiangsu University, Yixing, Jiangsu, China; 2 Graduate school, The Second Affiliated Hospital of Hainan Medical University, Haikou, Hainan, China

**Keywords:** blood sampling time deviation, logistic regression, therapeutic drug monitoring, trough concentration, vancomycin

## Abstract

**Objective:**

To evaluate the impact of deviations in blood sampling time on the interpretation of measured trough concentrations in vancomycin therapeutic drug monitoring; to explore a reference time window for early blood sampling in clinical settings where trough concentration monitoring remains the primary approach.

**Methods:**

A retrospective analysis was conducted on 160 therapeutic drug monitoring (TDM) records from inpatients receiving vancomycin TDM in our hospital between March 2024 and March 2026. Among these, 137 early-sampled cases were included for analysis, with predicted trough concentrations (C_pred) obtained using the Pharmado platform. Using C_pred as a model-assisted reference, the relative standard deviation (RSD) of measured concentrations (C_obs) was calculated. The target range of 10–20 μg/mL was applied to assess misjudgments in target attainment. Spearman correlation and Logistic regression analyses were employed to evaluate the relationship between time deviation (Δt) and RSD.

**Results:**

C_obs exhibited a systematic tendency toward overestimation compared to C_pred, with a significant positive correlation between Δt and RSD (*ρ* = 0.62, *p* < 0.001). In the group with sampling within 1 h before scheduled dosing, the median RSD was 10.8%, with 81.3% of cases meeting |RSD| ≤ 20% and zero misjudgments in target attainment. For sampling 1–2 h prior, this proportion declined to 60.0%. In the >2 h group, it fell to less than 24.6%, with significantly elevated misjudgment risk. Logistic regression confirmed advancing sampling time negatively predicted |RSD| ≤ 20% probability (*β*₁ = −1.088, odds ratio = 0.337). The model estimated that when blood sampling occurred approximately 0.83 and 2.10 h in advance, this probability decreased to 80 and 50%, respectively; however, these time points and the ≤1 h reference window should be considered exploratory findings conditional on the predictive performance of the Pharmado model and the limited sample size, and external validation is required before broader clinical application.

**Conclusion:**

Early blood sampling in vancomycin TDM can lead to systematic overestimation of trough concentrations, with greater deviations associated with longer early sampling times. When the blood sampling time is advanced by ≤1 h, the deviation between the measured concentration and the predicted trough concentration is minimal. Under the condition that the sampling time is accurately recorded, the measured value may be cautiously used for target concentration assessment. If the sampling time falls outside this window, either platform-based correction or re-sampling is recommended. The proposed 1-h threshold should be interpreted as an exploratory, model-dependent reference rather than a definitive clinical cutoff.

## 1 Introduction

Vancomycin is a critical antibiotic for treating infections caused by resistant gram-positive bacteria such as *Methicillin-resistant Staphylococcus Aureus* (MRSA). Due to its narrow therapeutic window and significant inter-individual pharmacokinetic variability, therapeutic drug monitoring (TDM) is essential to ensure its safe and effective use (1, 2). Current international guidelines recommend area under the curve over 24 h/minimum inhibitory concentration (AUC_24_/MIC) as the preferred exposure target for adult patients with severe MRSA infections (3), and relevant clinical studies also support AUC_24_/MIC as an efficacy-related exposure index. However, this strategy relies on Bayesian software and multi-point sampling, demanding higher technological platforms and personnel resources (4). Although the 2020 Chinese guidelines introduced the concept of AUC monitoring, they still retain the trough concentration indicator (target 10–20 μg/mL) (2, 5). Therefore, this study focuses on trough concentration monitoring, which does not deny the advantages of AUC-guided monitoring. Rather, it acknowledges that the vast majority of hospitals in China, especially primary hospitals, are currently unable to routinely perform AUC_24_/MIC monitoring. Under these circumstances, misjudgment of trough concentrations caused by early blood sampling remains one of the most common and manageable procedural issues in routine single-point TDM practice.

However, the interpretative value of trough concentration monitoring is highly dependent on the accuracy of the sampling time. Theoretically, trough samples should be collected immediately before the next dose. In real-world clinical practice, early blood sampling is common due to factors such as nursing shift schedules and the timing of medication order execution (6). Directly interpreting an early-measured concentration as the trough concentration can lead to misjudgment of target attainment, subsequently interfering with dose adjustment decisions. Current research on this issue is largely based on model simulations, and there is insufficient evidence regarding the permissible range of early sampling and the reliability of results in real-world settings (7).

In this context, this study, based on vancomycin TDM cases from our hospital, utilized actual sampling times and platform-predicted trough concentrations to analyze the impact of blood sampling time deviations on trough concentration interpretation and clinical decision-making. It also aimed to explore a feasible time window for early sampling, providing practical references for current clinical practice primarily relying on trough concentration monitoring.

## 2 Materials and methods

### 2.1 Study design and population

This was a single-center, retrospective observational study. Data were retrospectively collected from medical records of inpatients who received vancomycin therapy and underwent TDM in our hospital between March 1, 2024, and March 1, 2026. Leveraging the hospital’s tier-6 informatization closed-loop system, all included cases had complete and traceable records of medication administration and blood sampling times. Collected variables included: sex, age, height, weight, serum creatinine level, creatinine clearance (CrCl), dosing regimen, measured drug concentration (C_obs), theoretical sampling time, and actual sampling time.

This study was conducted in accordance with the ethical principles of the Declaration of Helsinki. The study protocol was reviewed and approved by the Ethics Committee of The Affiliated Yixing Hospital of Jiangsu University (Approval No. 202513701). Due to the retrospective, observational nature of the study and the exclusive use of de-identified patient data, the requirement for obtaining written or verbal informed consent was formally waived by the aforementioned ethics committee.

#### 2.1.1 Inclusion criteria

① Age ≥ 18 years; ② Received intravenous vancomycin during hospitalization; ③ Underwent TDM after reaching steady state; ④ Complete records of administration, sampling time, and predicted trough concentration data were available.

#### 2.1.2 Exclusion criteria

① Incomplete patient basic information; ② Not at steady state; incomplete administration or sampling time records; ③ Missing key variables; ④ Receiving renal replacement therapy; ⑤ Rapidly fluctuating renal function.

### 2.2 Data definitions

Blood Sampling Time Deviation: time deviation (Δt) was defined as actual sampling time−theoretical sampling time (unit: h). Δt < 0 indicates early sampling, Δt > 0 indicates delayed sampling.

Predicted Trough Concentration (C_pred): Theoretical trough concentrations for early-sampled samples were predicted using the Pharmado platform.^1^

Relative standard deviation (RSD) = (C_obs − C_pred) / C_pred × 100%.

Definition of patient type: Patients were classified as critical if they met either of the following criteria at the time of TDM sampling: (1) hospitalized in an intensive care unit; or (2) explicitly documented by the treating team as requiring critical care in the medical records. All other patients were classified as non-critical. This classification was performed retrospectively by the investigators based on medical records, ward location, and information provided on the TDM request form.

### 2.3 The Pharmado platform

All data processing and model construction were performed using the Pharmado platform (China). This platform is freely accessible web-based software. According to the publicly available instructions for use, the platform incorporates an adult vancomycin population pharmacokinetic model based on a two-compartment structure with first-order elimination. The model utilizes Bayesian feedback to individualize parameter estimates and includes covariates such as patient age, sex, body weight, serum creatinine, dosing regimen, and therapeutic drug monitoring results. The platform is intended for individualized vancomycin dosing design in adults, primarily serving as a reference for treatment, teaching, and research (8).

Acknowledging that the full parameterization and internal validation details of the platform are not publicly available, and that its external validity in this study cohort was not independently validated within this study, the platform’s output was solely used as a unified model-assisted reference to compare the directional deviation of C_obs relative to the theoretical trough concentration at different sampling time points, and was not regarded as the gold standard for true trough concentrations. To minimize the potential impact of model misspecification, platform-based extrapolation was applied only to early blood samples obtained from adults with regular dosing schedules and relatively stable renal function. The use of this platform was solely for research purposes, and the software provider had no role in study design, data analysis, interpretation of results, or manuscript preparation. Therefore, all quantitative inferences based on C_pred, including RSD, AUC estimation error, and the proposed sampling window, should be interpreted as conditional on the predictive performance of the Pharmado model in this study population.

### 2.4 Assessment of target attainment

In the assessment of target attainment, the model-assisted extrapolation results represented by C_pred were used as a unified reference standard, and classifications were made by comparing the target attainment status of C_obs with this reference. The target range was predefined as the therapeutic window of 10–20 μg/mL. True attainment: C_pred attained target, and C_obs attained target; False attainment: C_pred did not attain target, but C_obs attained target; False non-attainment: C_pred attained target, but C_obs did not attain target; True non-attainment: C_pred did not attain target, and C_obs did not attain target.

### 2.5 Exploration of acceptable time window

To quantify the impact of early sampling duration on the acceptability of concentration deviation, |RSD| ≤ 20% was defined as “numerically acceptable” (*Y* = 1), and |RSD| > 20% as “unacceptable” (*Y* = 0). The threshold value was defined based on two considerations. On one hand, it referred to the commonly accepted ±20% prediction error range used in external evaluations of previous vancomycin population pharmacokinetic models. On the other hand, considering that in decision-making scenarios involving trough concentration stratification, deviations exceeding 20% are more likely to alter the classification within the 10–20 mg/L target range and affect exposure extrapolation based on single-point concentrations, this study adopted it as the operational definition of acceptable error (9). Using the early sampling duration t = −Δt as the independent variable, a univariate logistic regression model was constructed:


$$\text{logit}(p)=\ln(\frac{p}{1-p})=\beta_{0}+\beta_{1}t$$

where p is the probability of |RSD| ≤ 20%. The regression coefficient, odds ratio OR = exp.(β_1_), and the model-predicted probability curve were reported. The thresholds of early sampling time corresponding to p dropping to 80 and 50% were calculated to define the reference window for interpreting early sampling results. The aforementioned threshold was used solely to describe a probability transition point and was not intended as a rigid clinical cutoff.

### 2.6 AUC estimation and error calculation

As a supplementary analysis, the potential impact of sampling-time deviation on AUC estimation was evaluated using two steady-state AUC_0–24_ estimates generated by the Pharmado platform under the same dosing history and patient covariates. First, the actual sampling time and C_obs were entered into the platform to obtain a model-estimated AUC that accounted for the true sampling time; this estimate was defined as the reference AUC (AUC_ref). Second, the platform-predicted trough concentration at the scheduled trough sampling time was used, together with the scheduled trough sampling time, to generate an AUC estimate based on the predicted trough concentration; this estimate was defined as AUC_pred. Of note, AUC_pred was derived from the same platform using its own predicted trough concentration, rather than from an independently measured value.

The relative AUC difference was calculated as (AUC_pred − AUC_ref)/AUC_ref × 100%. Importantly, neither AUC_ref nor AUC_pred represents an observed AUC derived from intensive pharmacokinetic sampling. Both estimates are model-assisted values generated by the Pharmado platform. Therefore, this supplementary analysis should be interpreted as a model-based comparison of AUC estimates derived from actual versus scheduled sampling information. The findings are conditional on the performance and assumptions of the Pharmado model.

### 2.7 Statistical analysis

SPSS 26.0 statistical software was used for data analysis. Normally distributed continuous variables were expressed as mean ± standard deviation, while skewed variables were presented as median. Given the markedly skewed distribution of Δt and the pharmacokinetic asymmetry between early and delayed sampling, this study focused on clinically frequent scenarios of early blood sampling. In correlation analysis, Spearman’s rank correlation was employed to assess the monotonic relationship between Δt and RSD, with the Spearman correlation coefficient (*ρ*) and two-tailed *p* value calculated. For regression analysis, only samples with Δt < 0 were included in univariate logistic regression fitting to mitigate interference from data skewness and directional differences on model interpretation. Additionally, due to the limited sample size in the ≤1 h early subgroup, which was insufficient to support robust multivariable analysis, the model results were primarily used to describe probability trends rather than as a basis for definitive inference.

## 3 Results

### 3.1 General characteristics

A total of 160 inpatients were included in this study, comprising 112 males (70%) and 48 females (30%). The median age was 71 years (interquartile range (IQR) 21–99 years), median weight was 63 kg (IQR 39–95 kg), median height was 165 cm (IQR 150–180 cm), and median BMI was 23.4 kg/m^2^ (IQR 14.2–32.6 kg/m^2^). The median serum creatinine was 58.4 μmol/L (IQR 19.8–206.8 μmol/L). Regarding dosing regimens, the most common single dose was 1.0 g (69.3%), and the most common dosing interval was q12h (85%), as shown in Table 1.

**Table 1:** Baseline characteristics of the study population.

Characteristic	Category	Value
Sex	Male	112 (70)
	Female	48 (30)
Age (years)		71 (21, 99)
Weight (kg)		63 (39, 95)
Height (cm)		165 (150, 180)
BMI (kg/m^2^)		23.4 (14.2, 32.6)
Serum creatinine (μmol/L)		58.4 (19.8, 206.8)
Creatinine clearance (mL/min)	>130	1(0.63)
	70–130	131(81.88)
	50–70	20(12.5)
	30–50	6(3.75)
	<30	2(1.25)
Patient type	Critical patients	29 (18.13)
	Non-critical patients	131 (81.87)
Single dose (g)	<0.5	4 (2.5)
	0.5	40 (25)
	0.5–1.0	2 (1.3)
	1	111 (69.3)
	>1.0	3 (1.8)
Dosing interval (h)	6	6 (3.7)
	8	11 (6.9)
	12	136 (85)
	24	7 (4.4)

Data are presented as median (IQR) or n (%).

### 3.2 Distribution of blood sampling time deviation (Δt)

**Figure 1:**
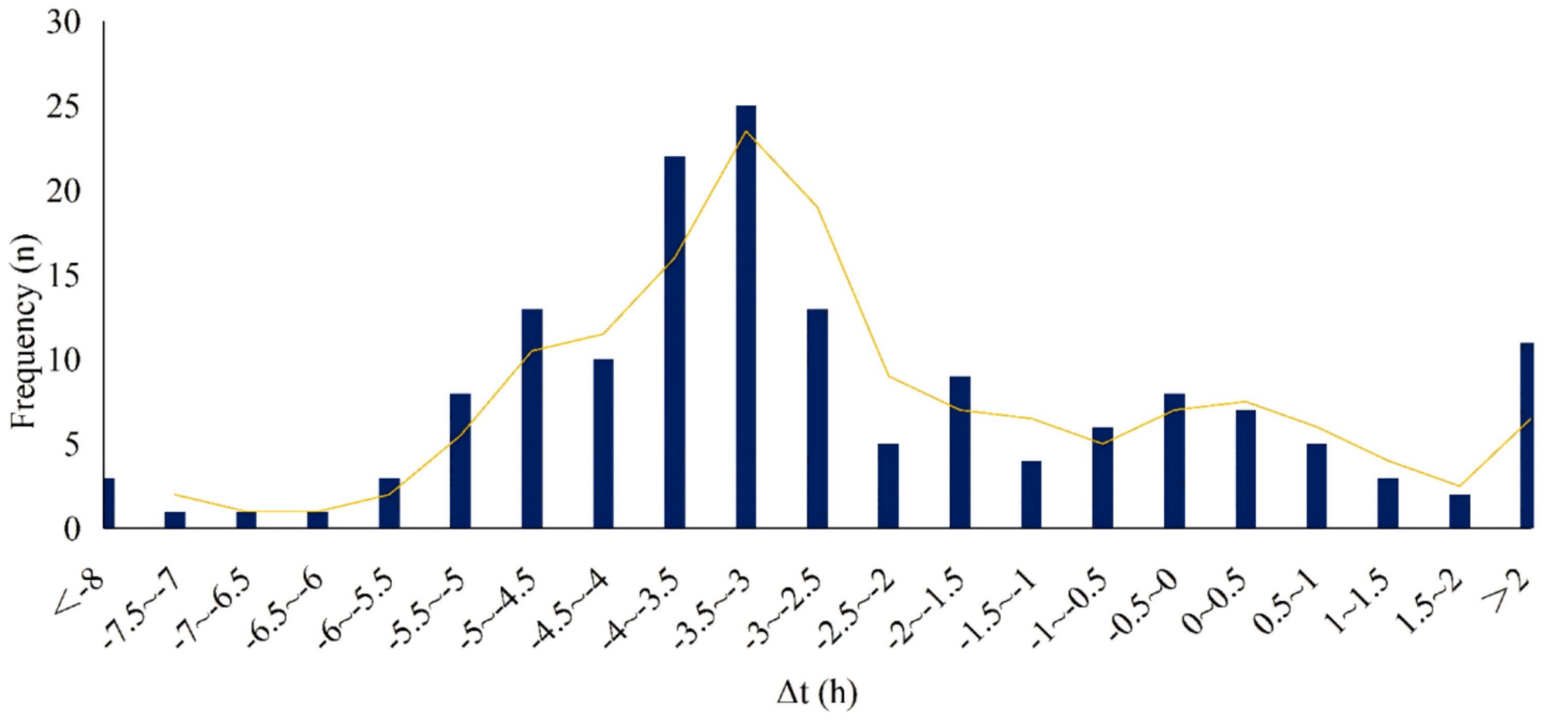
Distribution of Δt (*n* = 160).

The distribution characteristics of Δt in the study sample are shown in Figure 1. The distribution of Δt showed a distinct unimodal skew, with the peak concentrated in the interval of 3–4 h early (30%). Overall, early sampling predominated, accounting for 85.62%, while delayed sampling accounted for 14.38%, with few extreme deviations, suggesting a general trend toward early sampling in clinical practice. This distribution characteristic also provides data-based justification for focusing subsequent time window analyses on early sampling.

### 3.3 Relationship between Δt and concentration deviation

**Figure 2:**
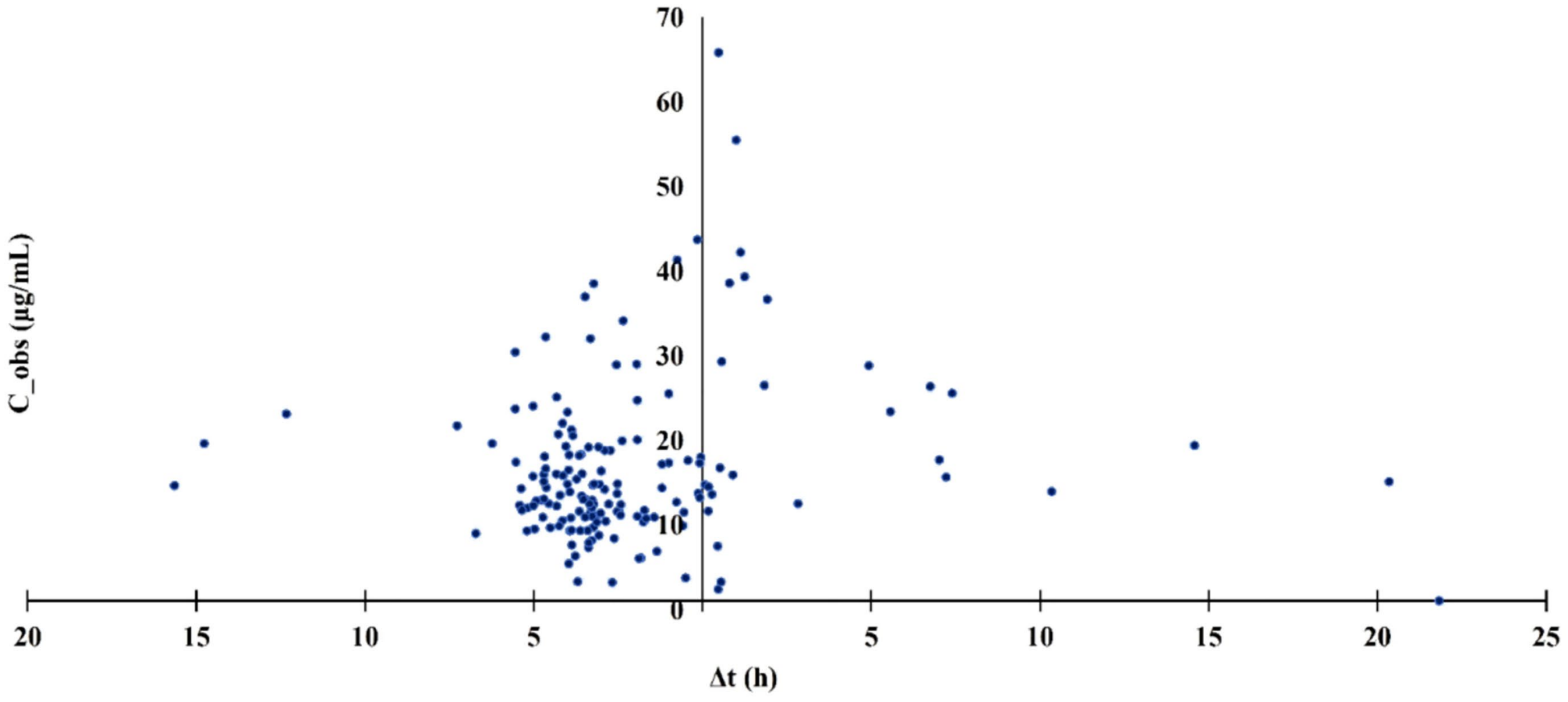
Scatter plot of C_obs vs. Δt (*n* = 160).

All samples (*n* = 160) had Δt values mainly concentrated in the negative range, and the corresponding measured concentrations showed a wide distribution with high dispersion, as shown in Figure 2. Given the extremely low explanatory power of the linear relationship between Δt and single C_obs values (*R*^2^ = 0.0075), the clinical informativeness of the linear fit was limited; therefore, interpretation was focused on the observed variability in concentration measurements and on the deviation between measured concentrations and model-predicted trough concentrations.

### 3.4 Target attainment status

Given the low proportion of delayed sampling in this cohort and its pharmacokinetic phase being asymmetric to that of early sampling, direct combined modeling with early samples would introduce interpretive bias. Therefore, this study conducted C_pred prediction and deviation analysis only on samples with early sampling. Additionally, to minimize the impact of extreme clearance status on the absolute time window, the primary analysis excluded patients with renal dysfunction requiring significant vancomycin dosage adjustments, i.e., those with creatinine clearance <50 or >130 mL/min (10).

**Figure 3:**
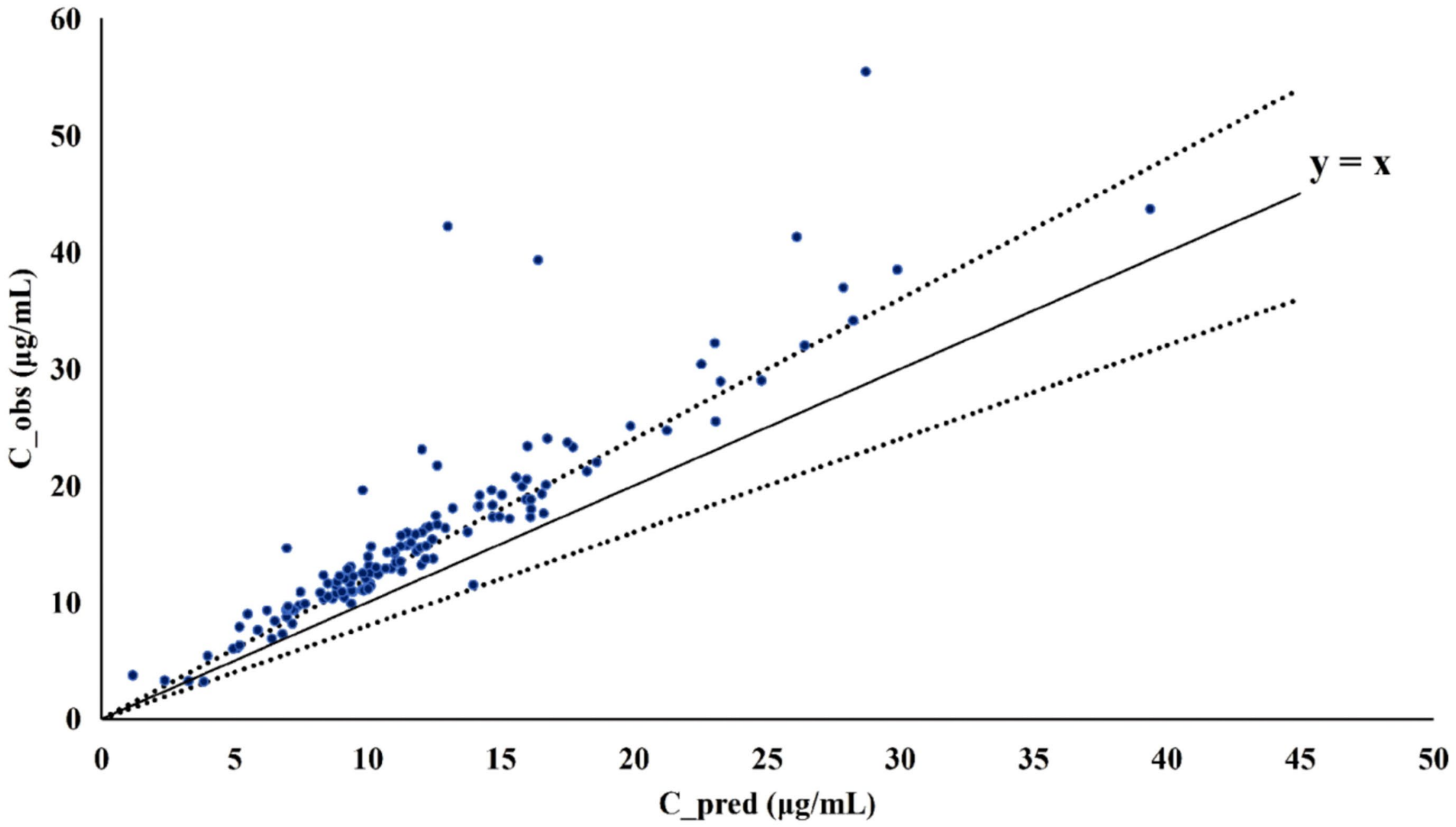
Scatter plot of C_obs vs. C_pred. C_obs was measured before the scheduled trough sampling time, whereas C_pred represents the predicted concentration at the scheduled trough time. Only samples with available platform-predicted values were included (*n* = 137). Solid line: y = x; dotted lines: ±20%.

Among the early-sampled samples with available platform predictions, using C_pred predicted by Pharmado as the reference standard, a scatter plot of C_obs vs. C_pred was generated to assess consistency, shown in Figure 3. Most points lie above the line of identity, indicating that measured concentrations tend to be higher than predicted trough concentrations overall. Using the 10–20 μg/mL target window for clinical interpretation classification, results are shown in Table 2. There were 58 cases (42.3%) of true attainment, 26 cases (19.0%) of false attainment, 14 cases (10.2%) of false non-attainment, and 39 cases (28.5%) of true non-attainment. This indicates that without correcting for sampling time deviation, measured concentrations can lead to a certain proportion of misinterpretations regarding target attainment, particularly manifesting as “false attainment.”

**Table 2:** Classification of clinical interpretation using target window (%).

C_pred/C_obs	C_obs attained	C_obs not attained	Total
C_pred attained	58 (42.33)	14 (10.22)	72
C_pred not attained	26 (18.98)	39 (28.47)	65
Total	84	53	137

### 3.5 Distribution of deviation of measured concentration from predicted trough concentration

In the early-sampled samples with available predicted trough concentrations, using C_pred as the reference, the RSD of measured concentrations was calculated. The RSD distribution was generally right-skewed, with predominantly positive values, indicating a systematic overestimation trend of C_obs compared to the theoretical trough concentration C_pred in cases of early sampling. The distribution was also relatively dispersed, suggesting significant inter-individual variability. Using |RSD| ≤ 20% as the acceptable error threshold, only 40/137 (29.20%) fell within this range, indicating that most early sampling results, if used directly as trough concentrations without time-based correction, could lead to significant deviations, providing an explanation for the observed misinterpretations of target attainment.

### 3.6 Relationship between Δt and RSD

In the early-sampled samples with available predicted trough concentrations, RSD was calculated using C_pred as the reference, and the degree of sampling deviation was quantified by early duration. Spearman rank correlation analysis showed a significant positive correlation between early duration and RSD (*ρ* = 0.62, *p* < 0.001), indicating that the earlier the sampling, the more pronounced the overestimation of measured concentration relative to the theoretical trough concentration.

Further stratification by Δt compared the deviation levels and clinical misjudgment risks across different degrees of earliness, as shown in Table 3. The results showed that in the group with sampling within 1 h before scheduled dosing (*n* = 16), the median RSD was 10.8% (IQR 7.1–16.4%), the proportion with |RSD| ≤ 20% was 81.3%, and no false attainment or false non-attainment was observed, suggesting high reliability of using C_obs for 10–20 μg/mL target interpretation within this time window. For sampling 1–2 h prior (*n* = 15), the median RSD increased to 19.0% (IQR 16.7–21.7%), the proportion with |RSD| ≤ 20% decreased to 60.0%, and a certain proportion of misjudgments were observed (false attainment 26.7%, false non-attainment 20.0%). In the group where the Δt is between −4 and −2 h (*n* = 65), the median RSD further increased to 25.9% (IQR 20.1–31.1%), with only 24.6% having |RSD| ≤ 20%. In the group with early sampling exceeding 4 h (*n* = 41), the median RSD reached 35.4% (IQR 30.9–39.9%), the proportion with |RSD| ≤ 20% dropped to 4.9%, and the rates of false attainment and false non-attainment increased to 29.3 and 19.5%, respectively, indicating a significantly increased risk when directly using C_obs as the trough concentration in this interval.

**Table 3:** RSD and misclassification risk stratified by early sampling duration.

Early sampling duration (h)	*n*	Median RSD (%)	Q1 (%)	Q3 (%)	|RSD| ≤ 20% (%)	False attainment (%)	False non-attainment (%)
≤ 1	16	10.8	7.1	16.4	81.3	0.0	0.0
1–2	15	19.0	16.7	21.7	60.0	26.7	20.0
2–4	65	25.9	20.1	31.1	24.6	15.4	4.6
>4	41	35.4	30.9	39.9	4.9	29.3	19.5

### 3.7 Definition of acceptable time window

**Figure 4:**
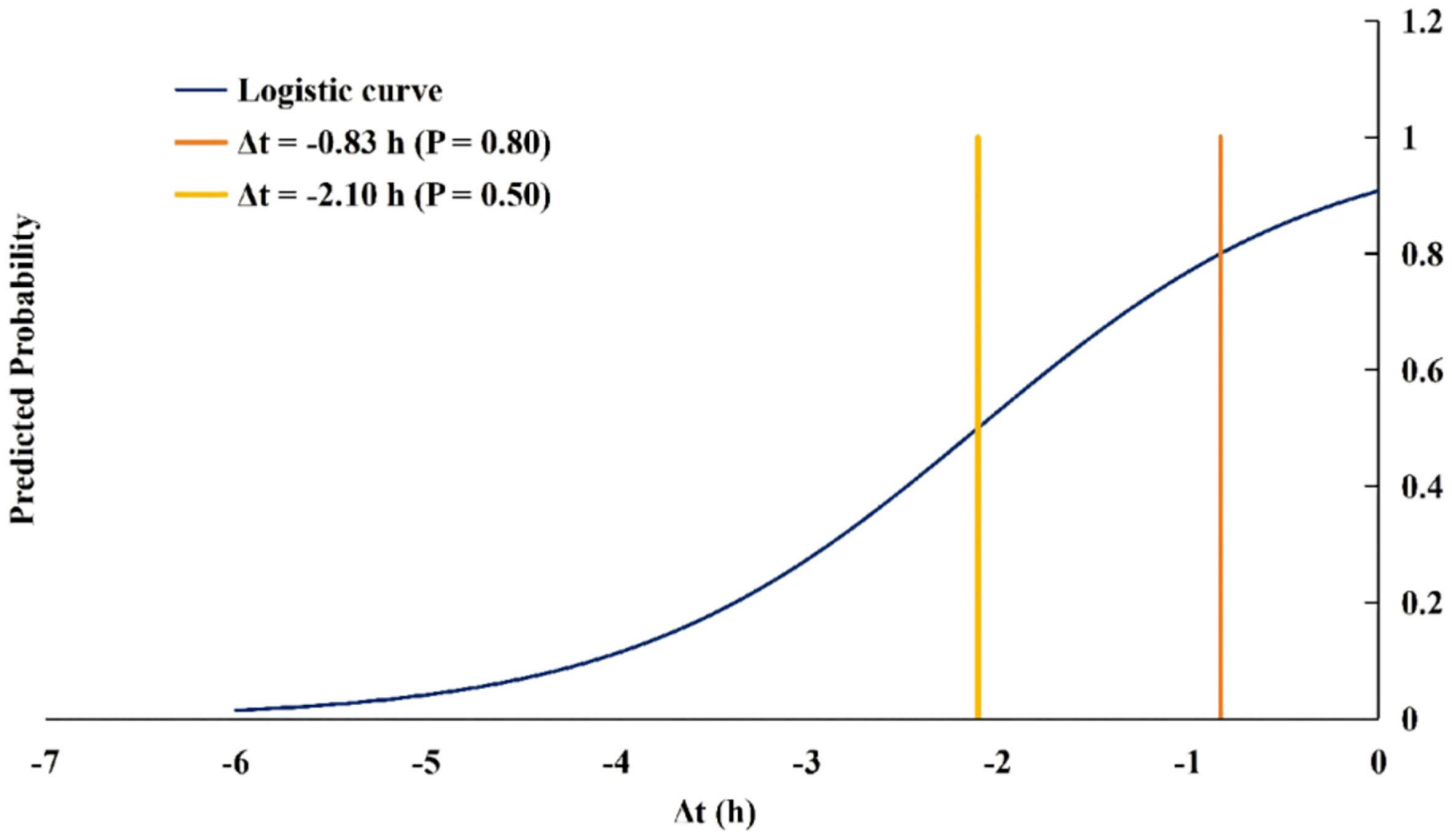
Logistic regression predicted probability curve of “|RSD| ≤ 20%” for different Δt and threshold definition (*n* = 137).

To quantitatively define the time window for “acceptable error,” a logistic model was constructed with |RSD| ≤ 20% as the outcome variable [logit(p) = 2.289–1.088·t, t = −Δt]. The results showed that the probability of this outcome decreased rapidly as the early sampling duration increased, as shown in Figure 4. The predicted advance time points corresponding to a decrease in the probability of |RSD| ≤ 20 to 80 and 50% were approximately 0.83 h and 2.10 h, respectively. However, given the limited sample size—particularly in the ≤ 1 h subgroup—these values are exploratory and model-dependent. Combining the stratification results and the probability model, this study suggests defining the reference window within which early sampling results can still be directly used for trough concentration target interpretation as early sampling ≤ 1 h. When sampling occurs more than 1 h early, it is recommended to obtain C_pred trough extrapolation based on the actual sampling time using Pharmado before dose adjustment, in order to reduce clinical decision-making biases caused by pseudo-target achievement or pseudo-suboptimal achievement.

### 3.8 Impact of sampling-time deviation on model-based AUC estimation

Based on the AUC_ref and AUC_pred values defined in the Methods section, the relative differences between model-based AUC estimates were compared across different sampling-time deviation intervals. As shown in Table 4, the median relative error of AUC difference decreased significantly as the sampling time approached the scheduled trough sampling time. When sampling was more than 4 h early, the median relative difference reached 25.6%, indicating that large sampling-time deviations may substantially affect model-based AUC estimation. When Δt was between −4 and −3 h, the median relative difference was 18.4%. When Δt was between −3 and −2 h, the median relative difference decreased to 9.2%, with most estimates showing smaller deviations. When Δt was between −2 and −1 h, the median error remained at a relatively low level of 8.7%. When Δt was within −1 h, the median relative difference was only 2.1%, and the range was markedly narrowed, suggesting that the impact of sampling time deviation on AUC estimation accuracy was minimized within this window.

**Table 4:** Relative error of AUC estimation across different Δt intervals.

Δt (h)	Sample size	Median relative difference (%)	Range (%)
< − 4	41	25.6	12.3–48.7
-4 ~ −3	47	18.4	5.6–31.2
−3 ~ −2	18	9.2	−3.1–22.8
−2 ~ −1	15	8.7	−4.5–21.3
−1 ~ 0	16	2.1	−0.5–4.7

## 4 Discussion and conclusion

This study, based on real-world TDM data, evaluated the impact of deviations in blood sampling time on the interpretation of vancomycin trough concentrations and subsequent clinical decision-making, focusing on clinical workflow errors rather than the dosing regimen itself. The results showed that early sampling is common in clinical practice; the early duration was significantly positively correlated with the RSD, and directly substituting the C_obs for the trough concentration led to systematic overestimation. Stratified analysis indicated that concentration bias and misjudgment risk increased stepwise with longer early durations, with early sampling ≥2 h leading to a significant decrease in the proportion of |RSD| ≤ 20%. Furthermore, this study constructed a probability curve for acceptable deviation using a logistic model, providing a quantitative basis for defining a reference window for sampling. It should be emphasized that, because the primary analyses relied on C_pred generated by Pharmado, the observed magnitude of bias and the derived time thresholds should not be interpreted as model-independent universal clinical cutoffs.

In real-world clinical workflows, trough concentration sampling tends to occur more frequently as early rather than delayed sampling, primarily due to factors such as nursing execution, medication order timing, and shift handovers. Therefore, while delayed sampling was included in data characterization, the derivation of the acceptable time window in this study was focused specifically on early sampling scenarios, aiming to address the most common sampling deviation issue that requires immediate management.

Our results also indicate that, in everyday clinical work, the share of vancomycin TDM samples drawn at the right time is still far from ideal. Hospitals should take this seriously and roll out focused training and quality-improvement work. For example, doctors, pharmacists, and nurses all need clearer instruction on when blood should be drawn in relation to dosing; prescribing and nursing systems can be set up to push reminders at the appropriate moment; specimen tubes should be labeled in a uniform way, with the actual draw time written down rather than assumed; and timely-sampling rates ought to be tracked and fed back to the wards on an ongoing basis. Doing these things should help staff stick more closely to the recommended sampling windows, and in turn cut down on situations where a vancomycin level is misread because the sample was taken at the wrong time.

From a pharmacokinetic perspective, early sampling captures a segment of the elimination phase that precedes the true trough, leading to systematic overestimation of concentration as the advance time increases (11). To minimize the impact of confounding factors such as dosing regimen and renal function, this study introduced a C_pred to construct RSD, making the directional bias caused by sampling time errors more observable. However, this approach has inherent limitations. The Pharmado platform employed in this study utilizes a proprietary population pharmacokinetic model. Although the model is based on a two-compartment structure with first-order elimination and incorporates covariates including patient age, sex, body weight, serum creatinine, dosing regimen, and TDM results, the full parameterization and internal validation details are not publicly available. Consequently, it remains challenging to determine whether the observed prediction errors are primarily attributable to deviations in sampling time documentation or to potential model misspecification. All results based on C_pred in this study should therefore be interpreted as model-assisted relative comparisons rather than determinations of absolute true values. This method cannot replace direct measurement of the actual trough concentration, nor can it eliminate systematic errors arising from population model mismatch. Accordingly, the proposed sampling window and all C_pred-based comparisons should be regarded as conditional on the performance of the Pharmado model in the present cohort.

Although international guidelines have recommended AUC_24_/MIC as the preferred target for vancomycin TDM, its estimation relies on multi-point sampling or Bayesian software, and methodological differences in MIC determination may also affect interpretation of the ratio, making it difficult to implement routinely in most local institutions at the current stage (12). In contrast, trough concentration measurement is simple, cost-effective, and has a more established workflow, remaining the primary monitoring approach in many Chinese hospitals. Therefore, this study does not pit trough concentration monitoring against AUC-guided monitoring; rather, it addresses the transitional clinical scenario where model-informed precision dosing (MIPD) has not yet been fully implemented, seeking to answer the practical question of whether a prematurely obtained single-point concentration can be directly interpreted (13). The value of this study lies primarily in providing a practical supplement to fill a gap in current practice, rather than serving as a methodological alternative.

Based on the risk of misinterpretation associated with varying degrees of deviation, a stratified management strategy is recommended. When sampling occurs ≤1 h before the scheduled dose, the deviation between C_obs and the predicted trough concentration is small; provided that the sampling time is accurately documented, the measured value can be used cautiously for target concentration assessment. Otherwise, platform-based correction or re-sampling is recommended. When early sampling exceeds 1 h, the likelihood of spurious results increases; C_obs should not be simply equated with the trough concentration, and correction via extrapolation using platforms such as Pharmado.net, incorporating the actual sampling time, is advisable. If early sampling reaches ≥2 h, the risk of misinterpretation is further amplified; reliance on platform-extrapolated C_pred or re-sampling at the standardized time is recommended. For early sampling exceeding 4 h, the measured value has very limited reference value, and direct re-sampling is strongly advised; platform-based extrapolation should be considered with caution only when both administration and sampling times are accurately recorded. It should be noted that from a methodological perspective, accurate recording of actual administration and sampling times coupled with extrapolation using MIPD/Bayesian models is superior to relying solely on fixed time windows. The time windows proposed in this paper are intended to offer a conservative, stratified strategy for scenarios where MIPD is not yet fully implemented, or where a rapid preliminary assessment of an early-collected sample is needed. In particular, the ≤1 h reference window was derived from a subgroup of only 16 patients and should currently be regarded as an indicative management threshold that requires confirmation through multicenter or external validation studies.

The absolute time thresholds derived from the logistic regression model in this study, such as 0.83 and 2.10 h, are inherently influenced by factors including clearance rate, half-life, dosing interval, and target concentration range, and thus exhibit significant population dependence. For populations with reduced clearance, such as those with chronic kidney disease, the same relative error may correspond to a wider absolute time window. Conversely, under conditions of augmented renal clearance, the tolerable absolute early sampling window may be narrower. Therefore, the thresholds provided in this paper are more suitable for interpreting findings in adult hospitalized populations similar to this study cohort and should not be mechanically extrapolated to individuals with significantly abnormal clearance. For such patient populations, priority should be given to MIPD/Bayesian extrapolation based on accurate sampling times, or the time windows should be recalibrated in future studies after stratification based on creatinine clearance or total clearance.

A strength of this study is the use of real-world closed-loop TDM data to examine sampling time deviations. Limitations include the single-center retrospective design, limited sample size in some subgroups, and the reliance on the proprietary Pharmado platform that was not independently validated. The AUC analysis was model-assisted, not measured. Future multicenter studies with closed-loop data are needed to validate these findings.

## Data Availability

The original contributions presented in the study are included in the article/supplementary material, further inquiries can be directed to the corresponding authors.

## Footnotes

1
http://www.pharmado.net
